# Insanity

**Published:** 1871-10

**Authors:** 


### INSANITY.

In former times, and recently, in some places in the United States and Great Britain, crazy persons were chained and kept in iron cages and out-houses in summer and winter. But now, large, commodious, and comfortable buildings are erected for the hapless insane; and instead of being beaten and bruised and cowed and tortured, they are treated with gentleness, tenderness, and consideration. The Pennsylvania Hospital for the insane, in Philadelphia, is one of the oldest in the country, and one of the most successful, as about half its inmates are sooner or later restored to health of mind and body too. It is conducted on the one great general principle of having varied and agreeable surroundings: varied, so as to compel the mind away from the one subject which has thrown it from its balance; and agreeable, so as to elevate and enliven, and inspire to better things than the indulgence of some one morbid passion or sentiment. To this great end, the utmost care and attention are paid in keeping the house tidy, light, airy, cheerful, and comfortable, winter and summer: the walls are adorned with engravings and paintings; some two dozen pianos are in constant use by the inmates; the grounds and gardens and fields connected with the building are elegantly laid out, both for useful work and exhilarating recreations; all this, under the admirable management of Dr. Kirkbride, who has for so many years labored assiduously and faithfully to bring the institution to its present high success.

Two prime practical facts present themselves in the history of this institution.

First, The sooner persons are sent there for treatment, after symptoms of insanity are observed by their friends, the greater certainty of a speedy and radical cure; if delayed over a year or two, recovery — permanent recovery — rarely, if ever, takes place.

Second, The more varied a person's employments are, the less likely he will be the inmate of a madhouse. That is, the man who has the most irons in the fire stands the best chance of having his wits about him to the last day of life. Look at the treadmill sameness of a farmer's or a merchant's life. In all lunatic asylums farmers are the most numerous, and merchants next; it is because one faculty of the brain is so constantly exercised, that an inflammatory action is set up, and that organ, in a measure, becomes disorganized at length; hence we see that lunatics are insane only on one subject, or class of subjects, while on all others they are quite rational, and on some, they exhibit extraordinary acuteness. Would the reader like to do something for these unfortunates, something to happify and brighten their hours of sadness, something to help cure them and return them to the circle of friends and home? then, when you have done with your picture-books and your illustrated weeklies, such as "Harper's," "Leslie's," and "Every Saturday," instead of laying them away on your shelves, with the intention of looking at them again, which you know you never do, or to have them bound at the end of the volume, to ornament your library, but never to be opened, send them post-paid to the nearest lunatic asylum, and you will be the cause of more happy hours and pleasant thoughts than can well be imagined.

The number of insane persons is increasing with fearful rapidity in all civilized countries; it is because, as society is now organized, the rich become richer, the poor poorer, and the strife for bread becomes more frantic; this produces unrest, anxiety, and carking, wearing care; and soon, the poor brain reels and gives way, and the man is mad. The remedy is, be content with moderate gains, get all the sleep you can, looking upward alway.

Three fourths of all the demented are from the class of persons whose absorbing aim in life has been to promote their own personal, individual aggrandizement; to carry out ends which centre in themselves — in painful economies for the sake of saving money, of hoarding it up. The liberal and generous are as one to thousands. For "the liberal soul" is made "fat."

